# Feasibility of Using Foot–Ground Clearance Biofeedback Training in Treadmill Walking for Post-Stroke Gait Rehabilitation

**DOI:** 10.3390/brainsci10120978

**Published:** 2020-12-13

**Authors:** Hanatsu Nagano, Catherine M. Said, Lisa James, Rezaul K. Begg

**Affiliations:** 1 Institute for Health and Sport (IHES), Victoria University, Melbourne, VIC 3011, Australia; Lisa.James@vu.edu.au (L.J.); rezaul.begg@vu.edu.au (R.K.B.); 2 Physiotherapy, Melbourne School of Health Sciences, The University of Melbourne, Melbourne, VIC 3053, Australia; csaid@unimelb.edu.au; 3 Physiotherapy Department, Western Health, St. Albans, VIC 3021, Australia; 4 Australian Institute for Musculoskeletal Science, St. Albans, VIC 3021, Australia; 5 Physiotherapy Department, Austin Health, Heidelberg, VIC 3084, Australia

**Keywords:** falls prevention, stroke rehabilitation, biofeedback gait training, tripping risk, minimum foot clearance

## Abstract

Hemiplegic stroke often impairs gait and increases falls risk during rehabilitation. Tripping is the leading cause of falls, but the risk can be reduced by increasing vertical swing foot clearance, particularly at the mid-swing phase event, minimum foot clearance (MFC). Based on previous reports, real-time biofeedback training may increase MFC. Six post-stroke individuals undertook eight biofeedback training sessions over a month, in which an infrared marker attached to the front part of the shoe was tracked in real-time, showing vertical swing foot motion on a monitor installed in front of the subject during treadmill walking. A target increased MFC range was determined, and participants were instructed to control their MFC within the safe range. Gait assessment was conducted three times: Baseline, Post-training and one month from the final biofeedback training session. In addition to MFC, step length, step width, double support time and foot contact angle were measured. After biofeedback training, increased MFC with a trend of reduced step-to-step variability was observed. Correlation analysis revealed that MFC height of the unaffected limb had interlinks with step length and ankle angle. In contrast, for the affected limb, step width variability and MFC height were positively correlated. The current pilot-study suggested that biofeedback gait training may reduce tripping falls for post-stroke individuals.

## 1. Introduction

Stroke is a major health risk, particularly among the aged population and, for example, more than 100 cases are reported daily in Australia [1]. Gait impairment is commonly associated with a stroke, but physical rehabilitation interventions can increase lower limb muscle strength and assist in re-establishing sensory-motor pathways [2]. In addition to improving balance and mobility in day-to-day activities, gait training may also be very important in reducing the risk of falling following in-patient rehabilitation [3]. The risk of falling is 150% higher (one-year post-stroke) in people with chronic stroke compared with age- and gender-matched controls [4]. In addition, approximately 50% of people living at home after a stroke will fall within 12 months [5], with up to half of these having multiple falls. It is, therefore, critically important to develop interventions to increase mobility and decrease the risk of falling. The problem addressed in this project was how to improve walking in stroke patients, with the aim of reducing their risk of tripping. 

Foot trajectory control can be seriously impaired by a stroke, leading to ankle weakness and reduced foot–ground clearance [6,7]. Tripping is recognised as the leading cause of falls [8,9], due to swing foot contact with the walking surface, or an object on it, with sufficient momentum to induce forward balance loss [10]. The critical gait event influencing the risk of tripping is Minimum Foot Clearance (MFC) at mid-swing, when the vertical margin between the lowest part of the foot and the walking surface is at its minimum [11,12]. In addition, the foot’s horizontal velocity is maximal at MFC, leading to highly forceful impact in the event of obstacle contact [12]. While increasing MFC height is, therefore, fundamental to preventing tripping, achieving consistent ground clearance, reflected in less MFC height variability, is also important for reducing tripping [11]. 

Lower and more variable MFC control over multiple steps and increased inter-limb asymmetry are typical in post-stroke individuals [13]. To address this problem, Begg and colleagues devised a biofeedback-based treadmill training procedure in which the trajectory of the forefoot marker motion was displayed in real time on a video monitor mounted in front of the treadmill [14,15]. While previous gait training for stroke patients has been designed to improve push-off forces (e.g., Liu et al. [16]), the protocol employed here, targeting foot–ground clearance to prevent tripping falls, was devised based on lower limb trajectory control research pioneered by Begg et al. [14].

The aim of the current study was to confirm the feasibility of MFC biofeedback training for post-stroke individuals and evaluate the practicality, safety and appropriateness of the training procedure. Furthermore, in this project we investigated in more detail how MFC control was achieved with practice, by examining the correlation between MFC and spatio-temporal gait parameters. The training intervention was hypothesised to improve foot trajectory control, shown operationally by (i) higher MFC, and (ii) less variable and (iii) more symmetrical MFC characteristics. If the training intervention promoted safer swing foot clearance, it was also of interest to examine how improved motor control is reflected in the coordination between the affected and unaffected limbs. 

## 2. Materials and Methods

### 2.1. Participants

There were seven post-stroke individuals over 18 years of age who had sustained a stroke within the previous six months but one withdrew due to constraints associated with travelling to the testing site, and therefore, six participants remained for analysis (Age: 68.0 ± 15.9 years; Height: 1.69 m ± 0.09 m; Body mass: 80.9 kg ± 19.2 kg), see also Table 1. They had no other health condition(s) that prevented them from walking on the treadmill, did not wear a foot orthosis to eliminate potential effects on foot clearance control, and were not planning to undertake physiotherapy during the study period. All were volunteers and they provided informed consent using procedures approved and mandated by the Austin Health Human Research Ethics Committee. The study conformed to CONSORT guidelines and was registered on the Australian and New Zealand Clinical Trials Registry (ANZCTR): ACTRN12613000286741.

### 2.2. Testing and Biofeedback Training Protocol

**Figure 1:**
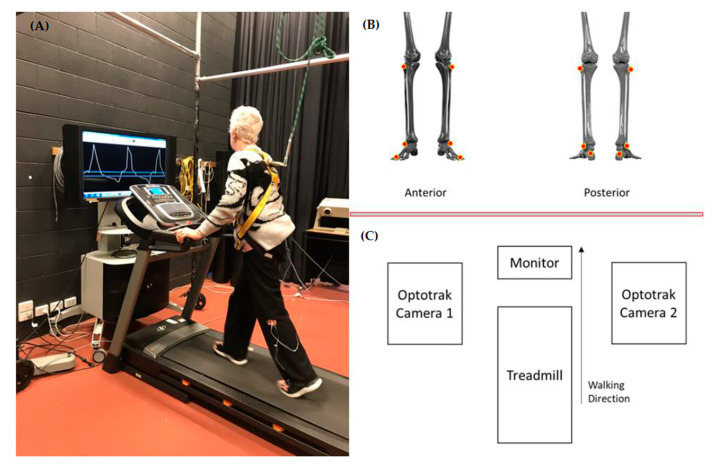
Experimental setup for biofeedback training. Two horizontal blue lines indicating target MFC range; a participant aiming to control MFC within the band to increase MFC height and improve consistent control. (**A**) Biofeedback training-only toe marker; (**B**) locations of IREDs attachment in gait assessment; (**C**) Experimental setup, two Optotrak cameras positioned bilaterally to cover the entire capture volume.

**Figure 2:**
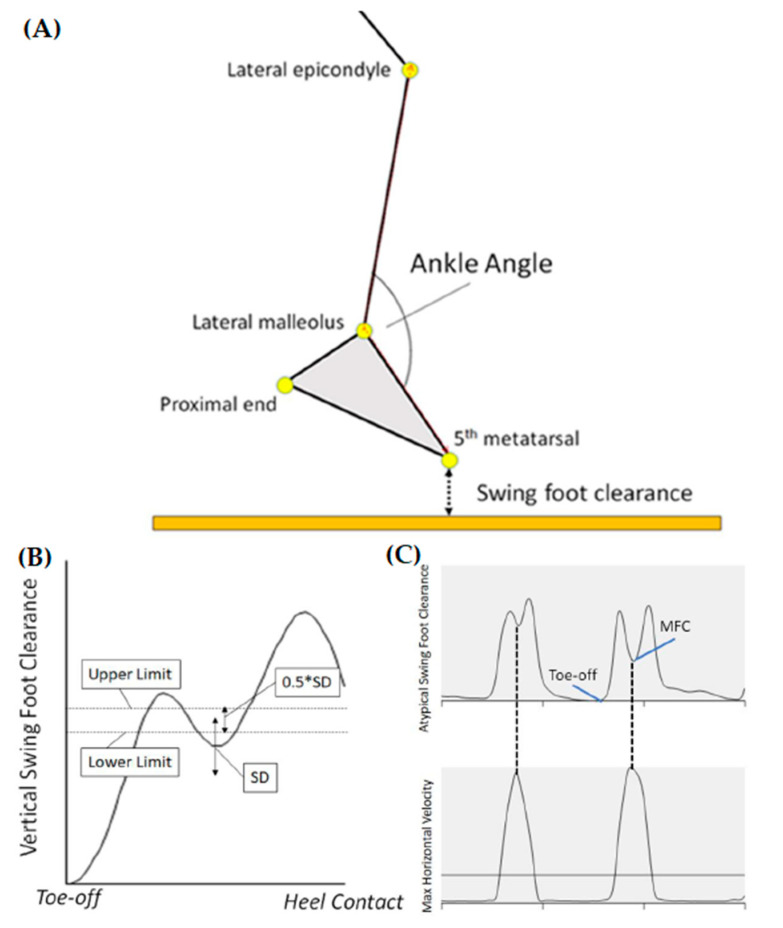
(**A**) Definitions of ankle angle (plantarflexion/dorsiflexion = increase/decrease) and swing foot clearance, increased/decreased ankle angle indicating plantarflexion/dorsiflexion; (**B**) A post-stroke individual’s swing foot clearance, target range between upper and lower limits; (**C**) Alternative definition of MFC based on maximum horizontal velocity of the swing foot.

Gait testing was conducted before (Baseline) eight training sessions over 4 weeks using the MFC biofeedback procedure (see Figure 1 and Figure 2B), followed by a post test (Post) on the final training day and a retention test 1 month later (1 Month). Preferred treadmill walking speed at Baseline was used throughout training and for the three gait assessment tests. All participants were equipped with a safety harness and used the handrails to maintain stability. At the beginning of the first session of biofeedback training, the baseline MFC data were sampled from the affected limb. Based on these baseline MFC data, target biofeedback training MFC for the affected limb was determined by using the following equation to compute upper and lower thresholds of the target MFC range (Figure 2B).

(Mean MFC+SD)±(0.5×SD)

As indicated in Figure 1A, two horizontal lines were, therefore, presented to indicate a subject-specific target MFC range, with the subject asked to maintain their MFC consistently within the band across all gait cycles during biofeedback training [14,15]. During the training sessions, participants were asked to maintain their affected limb MFC within the target bounds (Using only one marker on the toe of the affected limb). Each training lasted for up to 10 min depending on participants’ physical capacity. A retention test was scheduled one month after the Post assessment and participants were asked to maintain their daily lives without engaging in biofeedback training. 

### 2.3. Marker Setup and Data Analysis

For gait assessment, infrared emitting diodes (IREDs) were attached to the fifth metatarsal head (small toe), the proximal inferior surface of the shoe-outer sole (heel), lateral malleolus and lateral epicondyles of both feet (Figure 1B). Toe-off and heel contact were identified using standard gait analysis acceleration and velocity-based algorithms [14,15,17]. IREDs were sampled at 100 Hz using two Optotrak (Optotrak®, NDI, Canada) camera units positioned bilaterally to sample the treadmill walking area to sample the treadmill walking area to ensure an uninterrupted motion capture volume, as described in Figure 1C [11,14,15,17]. All obtained raw data were analysed using Visual 3D (C-motion), the general-purpose application for 3D movement analysis. The raw three-dimensional position-time data were first interpolated up to 10 frames (0.1 s) to compensate any occluded signals, and a 4th-order zero-lag Butterworth Filter with a cut-off frequency of 6 Hz was then applied [11].

### 2.4. Spatio-Temporal Step Parameters

Step length and width were determined, respectively, as anterior-posterior and medio-lateral displacement between the heels at heel-ground contact. Step time was the temporal interval from one heel contact to contralateral heel contact. 

### 2.5. Minimum Foot Clearance (MFC)

Post-stroke individuals may demonstrate atypical swing foot trajectories that do not show a conventionally defined MFC event and, in such circumstances, maximum swing foot horizontal velocity timing has been used to characterise occurrence of the MFC event [12]. This criterion was employed in the current study with vertical displacement at this time used to represent MFC height (Figure 2C). 

### 2.6. Ankle Angle

Ankle angle at MFC was formed by markers defining the lateral epicondyle, lateral malleolus and 5th metatarsal head (Figure 2A), with smaller angles indicating increased ankle dorsiflexion.

### 2.7. Design and Analysis

Due to the small sample size, nonparametric procedures were employed for analysis (SPSS 20.0, SPSS Inc., Chicago, IL, USA). An independent-samples Kruskal–Wallis Test was used to test whether obtained dependent variables were differentiated between the 6 classes: 3 conditions (Baseline, Post and 1 month) × 2 limbs (affected and unaffected). Spearman’s correlation analysis was conducted for unaffected and affected limbs separately for MFC (mean and SD) and stride phase variables to determine whether the affected and unaffected limbs would demonstrate different correlation patterns of foot clearance control. 

## 3. Results

**Figure 3:**
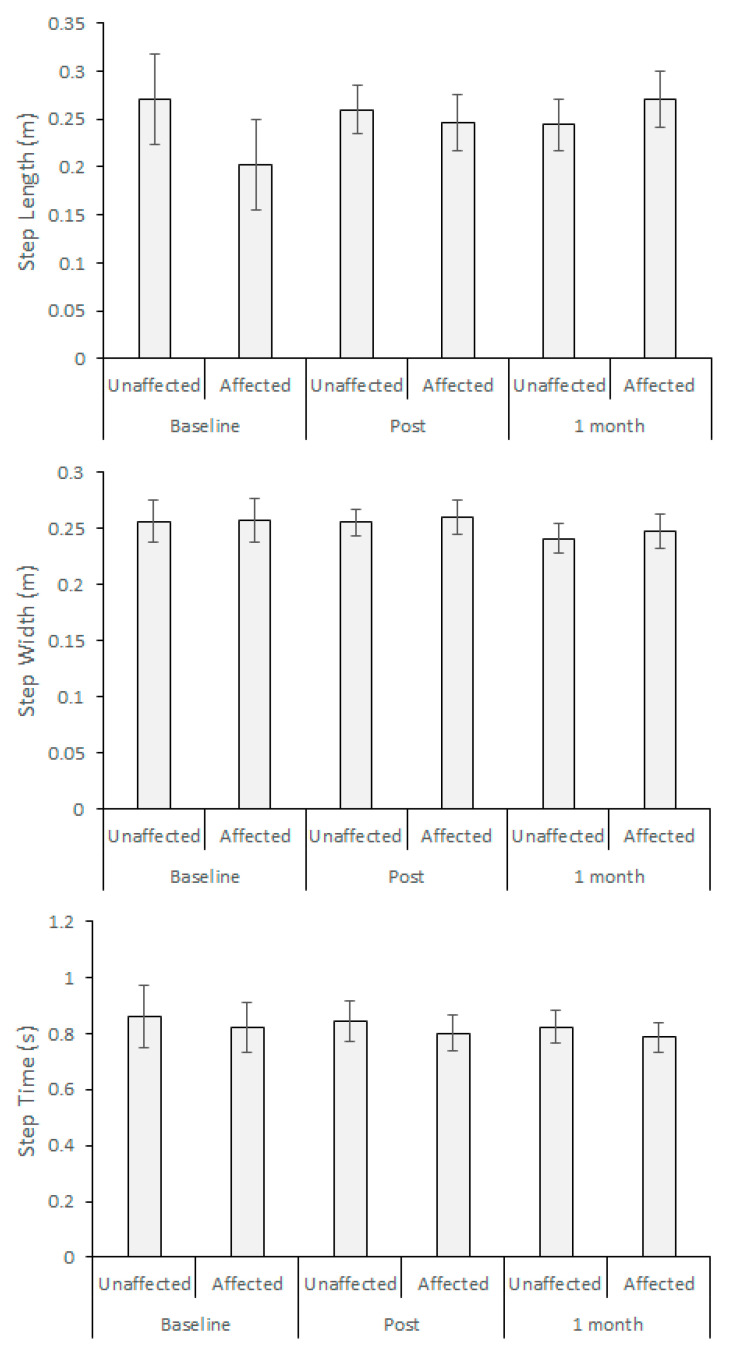
Spatio-temporal parameters: step length, step width and step time; Baseline, Post (post-training) and 1 Month (1 month after the post training).

As summarised in Figure 3, spatio-temporal gait parameters did not show differences between the affected and unaffected limbs. Training effects were, however, seen in step time variability due to a reduction from Baseline of 0.03 s and 0.05 s in the Post and 1-month tests, respectively, but no statistical significance was obtained. Similar trends in step length and step width variability were not statistically significant. 

**Figure 4:**
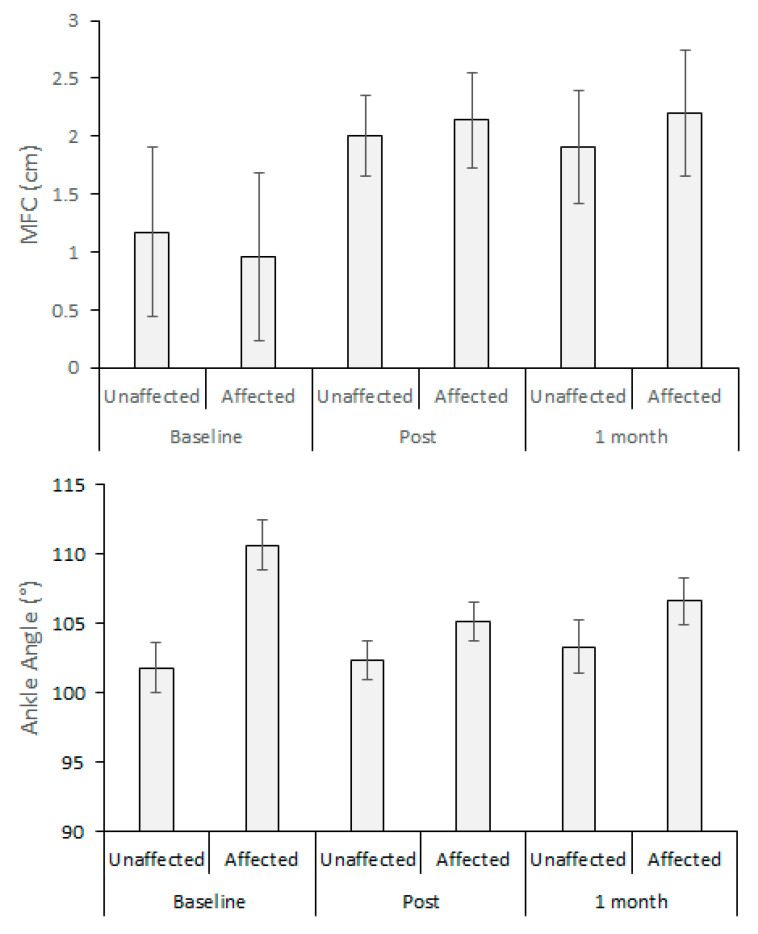
Minimum Foot Clearance (MFC); ankle angle (lower = dorsiflexion), Baseline, Post (post-training) and 1 Month (1 month after the post training).

As described in Figure 4, MFC data were distinguished (H (5) = 11.168, *p* < 0.05) due to the training effects in the affected limb, Baseline–Post (*p* < 0.05) and Baseline–1 month (*p* < 0.05) for 1.01 cm and 0.99 cm, respectively. As a result of biofeedback training, an overall reduction in MFC variability (SD) was also observed, but the effect did not attain significance. Contrary to the hypothesis, limb effects were not identified for MFC height or variability.

Ankle angle of the affected limb indicated 5.0° less dorsiflexion than the unaffected side, and especially at Baseline, the affected limb’s ankle angle was less dorsiflexed, but this trend was not statistically significant. 

For the unaffected limb, notable correlations were found between MFC and step length (r_s_ = 0.489, *p* < 0.05) and step length and step width (r_s_ = −0.541, *p* < 0.05). For the affected limb, in contrast, MFC was correlated with step width variability (r_s_ = −0.500, *p* < 0.05) and step length variability (r_s_ = 0.471, *p* < 0.05). The affected limb’s MFC variability was further correlated with step time (r = 0.666, *p* < 0.01).

## 4. Discussion

The primary aim of the biofeedback gait training in the current study was to assist post-stroke individuals with acquiring the precise end-point (i.e., toe) control required to maintain MFC within a safe range. Increased and less variable MFC was seen due to biofeedback training. While statistical significance was obtained only for increased MFC height, a trend of reduced MFC variability was, however, observed, and spatio-temporal gait parameters were also generally less variable following training, which can be attributed to the combined effects of MFC biofeedback and treadmill walking [17]. 

While some participants were relatively healthy and demonstrated less impaired gait, asymmetrical gait patterns were generally expected among post-stroke individuals. Contrary to expectation, the examined gait variables did not demonstrate asymmetry. Participants used the treadmill handrails such that their posture was more symmetrically aligned, possibly promoting more symmetrical gait. Gait characteristics have been shown to be more asymmetrical if individuals do not use the treadmill handrail [17,18], but this may not be generally feasible for post-stroke individuals, e.g., [19]. Treadmill walking with handrail support did, however, reduce gait variability including, critically, MFC variability. For future biofeedback training research, the current feasibility study suggests that the protocol of handrail use in treadmill walking should be standardised to reliably collect the data from a sufficiently large sample. Possibly due in part to sample size and lack of control group, spatio-temporal parameters did not show training effects, as in Figure 3, but further investigation is necessary with a larger sample size, including a control group, to examine whether there would really be no changes in spatio-temporal parameters (e.g., step length).

Despite generally symmetrical gait patterns, correlation analysis revealed different MFC control strategies for the affected and unaffected limbs. For the unaffected limb, increased step length contributed to higher MFC, consistent with previous studies [20,21]. In contrast, elevated MFC of the affected limb accompanied increased step length and width variability, indicating step-to-step adjustments to maintain stability [3,22]. Biofeedback gait training could, therefore, incorporate step length and width training to influence the highly correlated MFC by using the same visual real-time feedback procedures with a subject-specific target increased step length and/or width (Figure 1). Interestingly, both limbs showed high correlations between MFC variability and MFC height, suggesting that exaggerated attempts to increase swing foot clearance would be counterproductive by increasing MFC variability. 

Although a fully powered RCT is required for future studies to validate the obtained biofeedback effects, the current pilot research supports the hypothesis that biofeedback training can increase MFC and reduce gait variability of post-stroke individuals. The number of training sessions could be as few as eight per month, with the possibility of showing good retention of the targeted gait parameters one month following the final training session. For further understanding of how post-stroke individuals control swing toe movements, in-shoe foot pressure analysis can be another interesting approach to examine MFC control mechanics. Real-time foot pressure monitoring can be also possibly incorporated into biofeedback functions to enhance gait rehabilitation for stroke patients. Future studies should also focus on gait adaptations by the unaffected limb as the locomotive function of the affected side improves. Usually, the affected limb avoids risks and participates less in forward progression role [13], but improved gait function of the affected side should be reflected in the other limb to achieve symmetrical gait.

## Figures and Tables

**Table 1:** Characteristics of post-stroke individuals. SAH = subarachnoid haemorrhage, PICA = posterior inferior cerebellar artery, MCA = middle cerebral artery, ACA = anterior cerebral artery; STREAM = Stroke Rehabilitation Assessment of Movement.

Subject	Age (years)	Gait Speed (km/h)	Body Height (m)	Body Mass (kg)	Affected Limb	Time from Stroke (years)	Lesion Area	STREAM (20-Point Scale)
1	70	1.0	1.56	66.3	Right	21.0	SAH	13
2	80	1.1	1.70	78.8	Right	0.8	PICA	18
3	76	0.4	1.61	80.0	Left	1.4	MCA	14
4	67	1.5	1.80	115.1	Right	0.8	MCA	20
5	78	1.8	1.71	85.3	Left	0.7	ACA	16
6	37	1.3	1.77	59.7	Left	1.1	MCA	15
